# 2-Amino­benzoic acid–4-(pyridin-4-yl­disulfan­yl)pyridine (1/1)

**DOI:** 10.1107/S1600536811048483

**Published:** 2011-11-19

**Authors:** Hadi D. Arman, Trupta Kaulgud, Edward R. T. Tiekink

**Affiliations:** a Department of Chemistry, The University of Texas at San Antonio, One UTSA Circle, San Antonio, Texas 78249-0698, USA; b Department of Chemistry, University of Malaya, 50603 Kuala Lumpur, Malaysia

## Abstract

The title 1:1 co-crystal, C_7_H_7_NO_2_·C_10_H_8_N_2_S_2_, features a highly twisted 4-(pyridin-4-yldisulfan­yl)pyridine mol­ecule [dihedral angle between the pyridine rings = 89.06 (10)°]. A small twist is evident in the 2-amino­benzoic acid mol­ecule, with the C—C—C—O torsion angle being −7.7 (3)°. An N—H⋯O hydrogen bond occurs in the 2-amino­benzoic acid mol­ecule. In the crystal, mol­ecules are linked by O—H⋯N and N—H⋯N hydrogen bonds into a supra­molecular chain along the *b* axis. These are connected into layers by π–π inter­actions occurring between pyridine rings [centroid–centroid distance = 3.8489 (15) Å]. The layers are connected along the *a* axis by C—H⋯O contacts. The crystal studied was a racemic twin.

## Related literature

For related studies on co-crystal formation between carb­oxy­lic acids and pyridyl derivatives, see: Arman & Tiekink (2010 ▶); Wardell & Tiekink (2011 ▶); Arman *et al.* (2011 ▶).
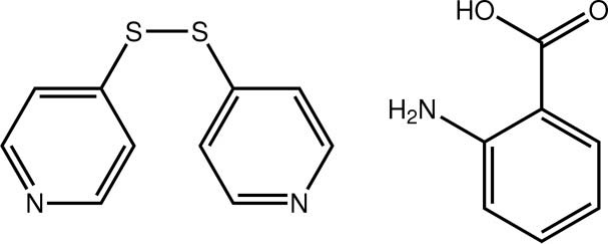


## Experimental

### 

#### Crystal data

C_7_H_7_NO_2_·C_10_H_8_N_2_S_2_
*M* *_r_* = 357.46
Monoclinic, 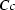

*a* = 8.636 (2) Å
*b* = 12.728 (3) Å
*c* = 15.688 (4) Å
β = 103.218 (4)°
*V* = 1678.7 (7) Å^3^
*Z* = 4
Mo *K*α radiation
μ = 0.33 mm^−1^
*T* = 98 K
0.30 × 0.27 × 0.15 mm

#### Data collection

Rigaku AFC12/SATURN724 CCD diffractometer
3149 measured reflections
3149 independent reflections
3115 reflections with *I* > 2σ(*I*)

#### Refinement

*R*[*F* ^2^ > 2σ(*F* ^2^)] = 0.031
*wR*(*F* ^2^) = 0.078
*S* = 1.03
3149 reflections
227 parameters
6 restraints
H atoms treated by a mixture of independent and constrained refinement
Δρ_max_ = 0.25 e Å^−3^
Δρ_min_ = −0.23 e Å^−3^
Absolute structure: nd
Flack parameter: ?
Rogers parameter: ?

### 

Data collection: *CrystalClear* (Molecular Structure Corporation & Rigaku, 2005 ▶); cell refinement: *CrystalClear*; data reduction: *CrystalClear*; program(s) used to solve structure: *SHELXS97* (Sheldrick, 2008 ▶); program(s) used to refine structure: *SHELXL97* (Sheldrick, 2008 ▶); molecular graphics: *ORTEPII* (Johnson, 1976 ▶) and *DIAMOND* (Brandenburg, 2006 ▶); software used to prepare material for publication: *publCIF* (Westrip, 2010 ▶).

## Supplementary Material

Crystal structure: contains datablock(s) global, I. DOI: 10.1107/S1600536811048483/zs2162sup1.cif

Structure factors: contains datablock(s) I. DOI: 10.1107/S1600536811048483/zs2162Isup2.hkl

Supplementary material file. DOI: 10.1107/S1600536811048483/zs2162Isup3.cml

Additional supplementary materials:  crystallographic information; 3D view; checkCIF report

## Figures and Tables

**Table 1:** Hydrogen-bond geometry (Å, °)

*D*—H⋯*A*	*D*—H	H⋯*A*	*D*⋯*A*	*D*—H⋯*A*
N1—H2*n*⋯O1	0.88 (2)	2.04 (2)	2.667 (2)	128 (2)
N1—H1*n*⋯N2^i^	0.88 (1)	2.15 (1)	3.027 (3)	173 (2)
O1—H1*o*⋯N3^ii^	0.84 (2)	1.79 (2)	2.621 (2)	173 (3)
C17—H17⋯O2^iii^	0.95	2.42	3.251 (3)	146

Symmetry codes: (i) 
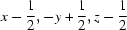
; (ii) 
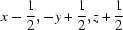
; (iii) 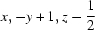.

**Fig. 1.**
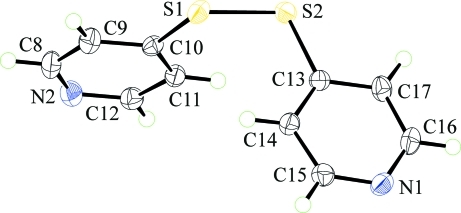
Molecular structure of 4-(pyridin-4-yldisulfanyl)pyridine in (I) showing atom-labelling scheme and displacement ellipsoids at the 50% probability level.

**Fig. 2.**
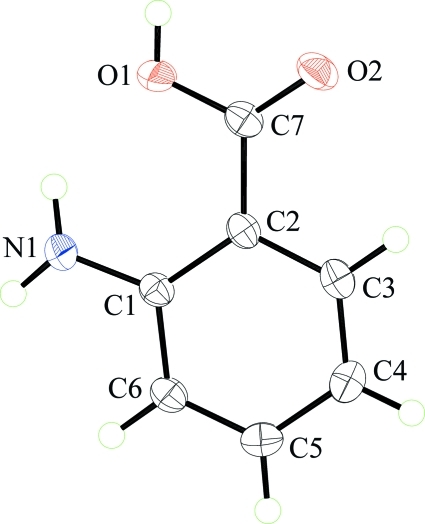
Molecular structure of the 2-aminobenzoic acid molecule in (I) showing atom-labelling scheme and displacement ellipsoids at the 50% probability level.

**Fig. 3.**
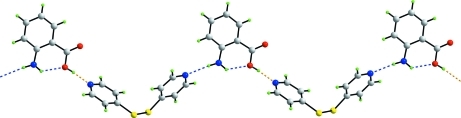
Supramolecular chain in (I) held together by O—H···N and N—H···N hydrogen bonds shown as orange and blue dashed lines, respectively.

**Fig. 4.**
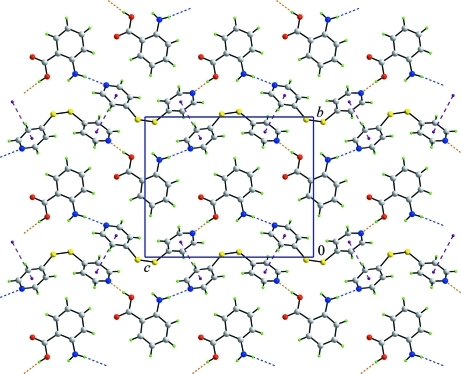
Supramolecular layer in (I) where the chains shown in Fig. 3 are linked by π–π interactions shown as purple dashed lines.

**Fig. 5.**
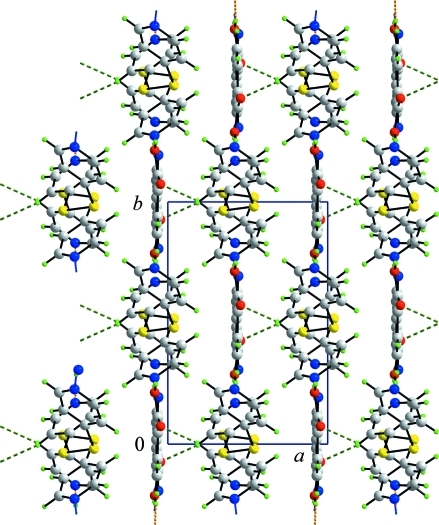
View in projection down the c axis of the unit-cell contents of (I), highlighting the C—H···O connections (green dashed lines) between the layers shown in Fig. 4.

## supplementary crystallographic information

### Comment

In connection with recent co-crystallization experiments of carboxylic acids with pyridyl-*N*-containing molecules (Arman & Tiekink, 2010; Wardell & Tiekink, 2011; Arman *et al.*, 2011), the 1:2 co-crystallization of 4-(pyridin-4-yldisulfanyl)pyridine and 2-aminobenzoic acid was investigated. This led to the isolation and characterization of the title 1:1 co-crystal, (I).

A single molecule of each of 4-(pyridin-4-yldisulfanyl)pyridine (Fig. 1), and 2-aminobenzoic acid (Fig. 2), comprise the crystallographic asymmetric unit of (I). The molecule is twisted with the 4-pyridyl rings being almost perpendicular to each other as seen in the value of the dihedral angle of 89.06 (10)°. The carboxylic acid residue is slightly twisted out of the plane of the benzene ring to which it is connected as seen in the C1—C2—C7—O1 torsion angle of -7.7 (3)°. This twist occurs despite the presence of an intramolecular N—H···O1 hydrogen bond (Table 1).

The most prominent feature of the crystal packing is the formation of supramolecular chains comprising alternating 4-(pyridin-4-yldisulfanyl)pyridine and 2-aminobenzoic acid molecules linked by O—H···N and N—H···N hydrogen bonds (Fig. 3 and Table 1). The chains pack into layers in the *bc* plane and are arranged so that pairs of chains face each other to allow for the formation of weak π–π interactions and for the interdigitation of the benzoic acid residues. The π–π interactions of 3.8489 (15) Å occur between the ring centroids of the (N2,C8–C12) and (N3,C13–C17)^iii^ pyridyl rings (Fig. 4) [symmetry code (iii) *x*, -*y* + 1, *z* + 1/2]. Layers stack along the *a* axis, being connected by C—H···O interactions [Fig. 5 and Table 1].

### Experimental

Colourless crystals of (I) were isolated from the 1:2 co-crystallization of 4-(pyridin-4-yldisulfanyl)pyridine (Sigma-Aldrich, 0.104 mmol) and 2-aminobenzoic acid (Sigma-Aldrich, 0.182 mmol) in chloroform solution (7 ml).

### Refinement

The C-bound H-atoms were placed in calculated positions (C—H = 0.95 Å) and were included in the refinement in the riding model approximation with *U*_iso_(H) set to 1.2*U*_eq_(C). The O– and N-bound H-atoms were located in a difference Fourier map and were refined with distance restraints of O—H = 0.840±0.001 Å and N—H = 0.880±0.001 Å, respectively, and with *U*_iso_(H) = 1.5*U*_eq_(O, N). The crystal studied was a racemic twin.

### Crystal data

**Table:**

C_7_H_7_NO_2_·C_10_H_8_N_2_S_2_	*F*(000) = 744
*M**_r_* = 357.46	*D*_x_ = 1.414 Mg m^−^^3^
Monoclinic, *C**c*	Mo *K*α radiation, λ = 0.71073 Å
Hall symbol: C -2yc	Cell parameters from 3807 reflections
*a* = 8.636 (2) Å	θ = 2.7–40.5°
*b* = 12.728 (3) Å	µ = 0.33 mm^−^^1^
*c* = 15.688 (4) Å	*T* = 98 K
β = 103.218 (4)°	Block, colourless
*V* = 1678.7 (7) Å^3^	0.30 × 0.27 × 0.15 mm
*Z* = 4	

### Data collection

**Table:**

Rigaku AFC12K/SATURN724 CCD diffractometer	3115 reflections with *I* > 2σ(*I*)
Radiation source: fine-focus sealed tube	*R*_int_ = 0.000
graphite	θ_max_ = 27.5°, θ_min_ = 2.7°
ω scans	*h* = −11→10
3149 measured reflections	*k* = 0→16
3149 independent reflections	*l* = −20→20

### Refinement

**Table:**

Refinement on *F*^2^	Secondary atom site location: difference Fourier map
Least-squares matrix: full	Hydrogen site location: inferred from neighbouring sites
*R*[*F*^2^ > 2σ(*F*^2^)] = 0.031	H atoms treated by a mixture of independent and constrained refinement
*wR*(*F*^2^) = 0.078	*w* = 1/[σ^2^(*F*_o_^2^) + (0.047*P*)^2^ + 0.5616*P*] where *P* = (*F*_o_^2^ + 2*F*_c_^2^)/3
*S* = 1.03	(Δ/σ)_max_ = 0.001
3149 reflections	Δρ_max_ = 0.25 e Å^−^^3^
227 parameters	Δρ_min_ = −0.23 e Å^−^^3^
6 restraints	Absolute structure: nd
Primary atom site location: structure-invariant direct methods	

### Special details

**Table:**

Geometry. All s.u.'s (except the s.u. in the dihedral angle between two l.s. planes) are estimated using the full covariance matrix. The cell s.u.'s are taken into account individually in the estimation of s.u.'s in distances, angles and torsion angles; correlations between s.u.'s in cell parameters are only used when they are defined by crystal symmetry. An approximate (isotropic) treatment of cell s.u.'s is used for estimating s.u.'s involving l.s. planes.
Refinement. Refinement of *F*^2^ against ALL reflections. The weighted *R*-factor *wR* and goodness of fit *S* are based on *F*^2^, conventional *R*-factors *R* are based on *F*, with *F* set to zero for negative *F*^2^. The threshold expression of *F*^2^ > 2σ(*F*^2^) is used only for calculating *R*-factors(gt) *etc*. and is not relevant to the choice of reflections for refinement. *R*-factors based on *F*^2^ are statistically about twice as large as those based on *F*, and *R*- factors based on ALL data will be even larger.

### Fractional atomic coordinates and isotropic or equivalent isotropic displacement parameters (Å^2^)

**Table:**

	*x*	*y*	*z*	*U*_iso_*/*U*_eq_	
S1	1.03842 (6)	0.51910 (4)	0.54403 (3)	0.02643 (12)	
S2	0.84697 (6)	0.53636 (3)	0.44236 (3)	0.02623 (12)	
O1	0.4176 (2)	0.28720 (11)	0.58052 (9)	0.0301 (3)	
H1o	0.425 (4)	0.2527 (19)	0.6269 (10)	0.045*	
O2	0.45445 (19)	0.42702 (12)	0.66806 (9)	0.0311 (3)	
N1	0.4410 (2)	0.30644 (14)	0.41463 (11)	0.0323 (4)	
H1n	0.428 (3)	0.2877 (17)	0.3594 (5)	0.048*	
H2n	0.418 (4)	0.2615 (14)	0.4525 (11)	0.048*	
N2	0.9169 (2)	0.27383 (14)	0.73043 (12)	0.0302 (4)	
N3	0.9212 (2)	0.33036 (13)	0.21812 (11)	0.0271 (3)	
C1	0.4350 (2)	0.41116 (14)	0.43227 (12)	0.0212 (4)	
C2	0.4369 (2)	0.45314 (14)	0.51627 (12)	0.0213 (4)	
C3	0.4360 (2)	0.56293 (16)	0.52675 (13)	0.0261 (4)	
H3	0.4382	0.5912	0.5831	0.031*	
C4	0.4322 (3)	0.63068 (16)	0.45784 (15)	0.0291 (4)	
H4	0.4322	0.7046	0.4665	0.035*	
C5	0.4282 (3)	0.58886 (16)	0.37511 (14)	0.0276 (4)	
H5	0.4246	0.6347	0.3269	0.033*	
C6	0.4294 (2)	0.48188 (15)	0.36270 (13)	0.0241 (4)	
H6	0.4263	0.4551	0.3058	0.029*	
C7	0.4379 (2)	0.38954 (15)	0.59532 (12)	0.0240 (4)	
C8	1.0466 (3)	0.33503 (17)	0.75021 (13)	0.0309 (4)	
H8	1.1159	0.3276	0.8064	0.037*	
C9	1.0850 (3)	0.40835 (17)	0.69325 (13)	0.0284 (4)	
H9	1.1792	0.4492	0.7096	0.034*	
C10	0.9828 (2)	0.42076 (14)	0.61162 (12)	0.0221 (4)	
C11	0.8478 (2)	0.35789 (15)	0.58888 (13)	0.0236 (4)	
H11	0.7764	0.3636	0.5332	0.028*	
C12	0.8218 (2)	0.28615 (15)	0.65130 (13)	0.0269 (4)	
H12	0.7298	0.2430	0.6363	0.032*	
C13	0.8862 (2)	0.45427 (14)	0.35820 (12)	0.0213 (4)	
C14	1.0171 (2)	0.38876 (15)	0.36659 (13)	0.0246 (4)	
H14	1.0959	0.3849	0.4198	0.030*	
C15	1.0288 (3)	0.32873 (16)	0.29402 (14)	0.0268 (4)	
H15	1.1186	0.2841	0.2989	0.032*	
C16	0.7979 (3)	0.39530 (17)	0.21060 (13)	0.0293 (4)	
H16	0.7221	0.3981	0.1562	0.035*	
C17	0.7749 (3)	0.45889 (16)	0.27830 (13)	0.0264 (4)	
H17	0.6857	0.5045	0.2705	0.032*	

### Atomic displacement parameters (Å^2^)

**Table:**

	*U*^11^	*U*^22^	*U*^33^	*U*^12^	*U*^13^	*U*^23^
S1	0.0318 (3)	0.0275 (2)	0.0204 (2)	−0.0070 (2)	0.00692 (19)	−0.00252 (17)
S2	0.0341 (3)	0.02419 (19)	0.0212 (2)	0.00549 (19)	0.00813 (19)	0.00134 (17)
O1	0.0455 (9)	0.0265 (7)	0.0172 (6)	−0.0018 (6)	0.0050 (6)	0.0027 (5)
O2	0.0382 (9)	0.0363 (8)	0.0188 (7)	−0.0070 (7)	0.0064 (6)	−0.0036 (6)
N1	0.0531 (12)	0.0238 (8)	0.0201 (8)	0.0013 (8)	0.0088 (8)	−0.0032 (6)
N2	0.0332 (10)	0.0307 (9)	0.0260 (9)	0.0007 (8)	0.0054 (7)	0.0039 (7)
N3	0.0332 (9)	0.0268 (8)	0.0216 (8)	−0.0033 (7)	0.0066 (7)	−0.0034 (7)
C1	0.0197 (8)	0.0249 (8)	0.0181 (8)	0.0014 (7)	0.0023 (7)	0.0000 (7)
C2	0.0188 (8)	0.0256 (8)	0.0185 (9)	−0.0009 (7)	0.0024 (7)	−0.0031 (7)
C3	0.0270 (9)	0.0290 (9)	0.0223 (9)	−0.0009 (8)	0.0053 (8)	−0.0054 (8)
C4	0.0306 (11)	0.0222 (9)	0.0334 (11)	0.0015 (8)	0.0053 (9)	−0.0014 (8)
C5	0.0282 (10)	0.0287 (9)	0.0253 (10)	0.0008 (8)	0.0050 (8)	0.0060 (8)
C6	0.0240 (10)	0.0291 (9)	0.0181 (9)	0.0001 (7)	0.0025 (8)	−0.0005 (7)
C7	0.0210 (9)	0.0301 (9)	0.0201 (9)	−0.0019 (7)	0.0030 (7)	−0.0010 (7)
C8	0.0307 (10)	0.0391 (11)	0.0207 (10)	0.0028 (9)	0.0011 (8)	0.0023 (8)
C9	0.0249 (9)	0.0349 (10)	0.0244 (10)	−0.0004 (8)	0.0033 (8)	−0.0007 (8)
C10	0.0245 (9)	0.0234 (9)	0.0193 (8)	0.0013 (7)	0.0068 (7)	−0.0030 (7)
C11	0.0233 (9)	0.0259 (8)	0.0209 (9)	0.0013 (7)	0.0035 (7)	−0.0009 (7)
C12	0.0290 (10)	0.0247 (9)	0.0267 (10)	−0.0017 (7)	0.0057 (8)	−0.0008 (7)
C13	0.0270 (10)	0.0199 (8)	0.0190 (9)	−0.0005 (7)	0.0092 (7)	0.0015 (6)
C14	0.0283 (10)	0.0246 (8)	0.0203 (9)	−0.0008 (7)	0.0043 (8)	0.0000 (7)
C15	0.0291 (10)	0.0258 (9)	0.0257 (10)	0.0015 (8)	0.0069 (8)	0.0004 (7)
C16	0.0319 (10)	0.0362 (10)	0.0185 (9)	−0.0031 (8)	0.0031 (8)	0.0025 (8)
C17	0.0276 (10)	0.0319 (9)	0.0202 (9)	0.0027 (8)	0.0068 (8)	0.0047 (7)

### Geometric parameters (Å, °)

**Table:**

S1—C10	1.7762 (19)	C4—H4	0.9500
S1—S2	2.0297 (8)	C5—C6	1.376 (3)
S2—C13	1.7761 (18)	C5—H5	0.9500
O1—C7	1.328 (2)	C6—H6	0.9500
O1—H1o	0.8399 (10)	C8—C9	1.384 (3)
O2—C7	1.215 (2)	C8—H8	0.9500
N1—C1	1.365 (2)	C9—C10	1.388 (3)
N1—H1n	0.8800 (11)	C9—H9	0.9500
N1—H2n	0.8801 (10)	C10—C11	1.391 (3)
N2—C12	1.332 (3)	C11—C12	1.394 (3)
N2—C8	1.341 (3)	C11—H11	0.9500
N3—C16	1.332 (3)	C12—H12	0.9500
N3—C15	1.332 (3)	C13—C14	1.387 (3)
C1—C6	1.407 (3)	C13—C17	1.396 (3)
C1—C2	1.418 (2)	C14—C15	1.394 (3)
C2—C3	1.407 (3)	C14—H14	0.9500
C2—C7	1.479 (2)	C15—H15	0.9500
C3—C4	1.377 (3)	C16—C17	1.385 (3)
C3—H3	0.9500	C16—H16	0.9500
C4—C5	1.396 (3)	C17—H17	0.9500
			
C10—S1—S2	105.22 (7)	N2—C8—H8	118.2
C13—S2—S1	105.12 (7)	C9—C8—H8	118.2
C7—O1—H1o	112 (2)	C8—C9—C10	118.5 (2)
C1—N1—H1n	117.5 (15)	C8—C9—H9	120.8
C1—N1—H2n	118.2 (15)	C10—C9—H9	120.8
H1n—N1—H2n	119.4 (18)	C9—C10—C11	119.33 (18)
C12—N2—C8	116.85 (18)	C9—C10—S1	115.41 (15)
C16—N3—C15	117.96 (17)	C11—C10—S1	125.26 (16)
N1—C1—C6	117.63 (17)	C10—C11—C12	117.17 (19)
N1—C1—C2	124.29 (18)	C10—C11—H11	121.4
C6—C1—C2	118.07 (17)	C12—C11—H11	121.4
C3—C2—C1	118.91 (17)	N2—C12—C11	124.59 (19)
C3—C2—C7	116.40 (16)	N2—C12—H12	117.7
C1—C2—C7	124.69 (17)	C11—C12—H12	117.7
C4—C3—C2	121.99 (18)	C14—C13—C17	119.32 (17)
C4—C3—H3	119.0	C14—C13—S2	124.98 (16)
C2—C3—H3	119.0	C17—C13—S2	115.70 (15)
C3—C4—C5	118.82 (18)	C13—C14—C15	117.50 (19)
C3—C4—H4	120.6	C13—C14—H14	121.2
C5—C4—H4	120.6	C15—C14—H14	121.3
C6—C5—C4	120.63 (18)	N3—C15—C14	123.7 (2)
C6—C5—H5	119.7	N3—C15—H15	118.1
C4—C5—H5	119.7	C14—C15—H15	118.1
C5—C6—C1	121.56 (18)	N3—C16—C17	123.1 (2)
C5—C6—H6	119.2	N3—C16—H16	118.4
C1—C6—H6	119.2	C17—C16—H16	118.4
O2—C7—O1	122.14 (18)	C16—C17—C13	118.30 (19)
O2—C7—C2	123.34 (18)	C16—C17—H17	120.8
O1—C7—C2	114.52 (16)	C13—C17—H17	120.8
N2—C8—C9	123.6 (2)		
			
C10—S1—S2—C13	−95.20 (9)	C8—C9—C10—C11	−1.5 (3)
N1—C1—C2—C3	177.7 (2)	C8—C9—C10—S1	178.27 (16)
C6—C1—C2—C3	−1.1 (3)	S2—S1—C10—C9	−169.32 (13)
N1—C1—C2—C7	−3.1 (3)	S2—S1—C10—C11	10.46 (18)
C6—C1—C2—C7	178.12 (18)	C9—C10—C11—C12	1.1 (3)
C1—C2—C3—C4	0.5 (3)	S1—C10—C11—C12	−178.70 (14)
C7—C2—C3—C4	−178.81 (18)	C8—N2—C12—C11	−0.3 (3)
C2—C3—C4—C5	0.3 (3)	C10—C11—C12—N2	−0.1 (3)
C3—C4—C5—C6	−0.5 (3)	S1—S2—C13—C14	3.90 (18)
C4—C5—C6—C1	−0.2 (3)	S1—S2—C13—C17	−175.51 (13)
N1—C1—C6—C5	−177.9 (2)	C17—C13—C14—C15	−1.2 (3)
C2—C1—C6—C5	1.0 (3)	S2—C13—C14—C15	179.40 (15)
C3—C2—C7—O2	−7.8 (3)	C16—N3—C15—C14	1.9 (3)
C1—C2—C7—O2	172.92 (19)	C13—C14—C15—N3	−0.6 (3)
C3—C2—C7—O1	171.55 (19)	C15—N3—C16—C17	−1.5 (3)
C1—C2—C7—O1	−7.7 (3)	N3—C16—C17—C13	−0.2 (3)
C12—N2—C8—C9	−0.2 (3)	C14—C13—C17—C16	1.6 (3)
N2—C8—C9—C10	1.1 (3)	S2—C13—C17—C16	−178.97 (15)

### Hydrogen-bond geometry (Å, °)

**Table:**

*D*—H···*A*	*D*—H	H···*A*	*D*···*A*	*D*—H···*A*
N1—H2n···O1	0.88 (2)	2.04 (2)	2.667 (2)	128.(2)
N1—H1n···N2^i^	0.88 (1)	2.15 (1)	3.027 (3)	173.(2)
O1—H1o···N3^ii^	0.84 (2)	1.79 (2)	2.621 (2)	173 (3)
C17—H17···O2^iii^	0.95	2.42	3.251 (3)	146

Symmetry codes: (i) *x*−1/2, −*y*+1/2, *z*−1/2; (ii) *x*−1/2, −*y*+1/2, *z*+1/2; (iii) *x*, −*y*+1, *z*−1/2.
